# Pancreatic adenocarcinoma exerts systemic effects on the peripheral blood myeloid and plasmacytoid dendritic cells: an indicator of disease severity?

**DOI:** 10.1186/1471-2407-10-87

**Published:** 2010-03-09

**Authors:** Vegard Tjomsland, Per Sandström, Anna Spångeus, Davorka Messmer, Johan Emilsson, Ursula Falkmer, Sture Falkmer, Karl-Eric Magnusson, Kurt Borch, Marie Larsson

**Affiliations:** 1 Division of Molecular Virology, Department of Clinical and Experimental Medicine, Linköping University, Sweden; 2 Division of Surgery, Linköping University, Sweden; 3 Division of Internal Medicine, Department of Medical and Health Science, Linköping University, Sweden; 4 Moores Cancer Institute, University of California, USA; 5 Department of Oncology and Radiotherapy, Jönköping Hospital, Sweden; 6 Morphology/Pathology Unit, Department of Laboratory Medicine, Jönköping Hospital, Sweden; 7 Division of Medical Microbiology, Department of Clinical and Experimental Medicine, Linköping University, Sweden

## Abstract

**Background:**

Dendritic cells (DCs) isolated from tumor bearing animals or from individuals with solid tumors display functional abnormalities and the DC impairment has emerged as one mechanism for tumor evasion from the control of the immune system. Ductal pancreatic adenocarcinoma (PDAC), the most common pancreatic cancer, is recognized as a very aggressive cancer type with a mortality that almost matches the rate of incidence.

**Methods:**

We examined the systemic influence ductal pancreatic adenocarcinoma (PDAC) exerted on levels of peripheral blood DCs and inflammatory mediators in comparison to the effects exerted by other pancreatic tumors, chronic pancreatitis, and age-matched controls.

**Results:**

All groups examined, including PDAC, had decreased levels of myeloid DCs (MDC) and plasmacytoid DCs (PDC) and enhanced apoptosis in these cells as compared to controls. We found elevated levels of PGE2 and CXCL8 in subjects with PDAC, and chronic pancreatitis. Levels of these inflammatory factors were in part restored in PDAC after tumor resection, whereas the levels of DCs were impaired in the majority of these patients ~12 weeks after tumor removal. Our results prove that solid pancreatic tumors, including PDAC, systemically affect blood DCs. The impairments do not seem to be tumor-specific, since similar results were obtained in subjects with chronic pancreatitis. Furthermore, we found that PDAC patients with a survival over 2 years had significant higher levels of blood DCs compared to patients with less than one year survival.

**Conclusions:**

Our findings points to the involvement of inflammation in the destruction of the blood MDCs and PDCs. Furthermore, the preservation of the blood DCs compartment in PDAC patients seems to benefit their ability to control the disease and survival.

## Background

Pancreatic duct adenocarcinoma (PDAC) is a lethal human cancer, with a five year survival rate of less than 5% [1,2]. PDAC is the tenth most common cancer, representing about 2% [3] of all cases of cancer, the grim prognosis makes it the number four when it comes to cancer deaths in the western world [2-4]. Despite all research efforts during the last 50 years, there are still no effective therapies for PDAC, except for surgical resection which has a minor impact on the long term survival rate [5]. Consequently, it is of great importance to acquire a deeper knowledge about the development and progression of PDAC in order to develop new treatment strategies for this aggressive cancer.

Increasing evidence points to a systemic impairment of the immune system in individuals with different types of cancers [6-8] putatively promoting tumor progression and development. Dendritic cells (DCs) are professional antigen presenting cells equipped for activation of naïve T cells and central memory T cells [9,10]. The DCs are ubiquitously distributed within the body and constitute less than 1% of peripheral blood mononuclear cells (PBMCs) [11,12]. Two distinct subtypes of DCs exist in the peripheral blood, i.e. the myeloid DCs (MDCs) and plasmacytoid DCs (PDCs). They share several common features, such as the expression of high levels of MHC class II molecules (HLA-DR) and lack of lineage specific markers (CD3, CD14, CD16, CD19, CD20, and CD56) [13]. MDCs express high levels of CD11c, BDCA1, and BDCA3 and myeloid related surface molecules, whereas PDCs lack the myeloid markers including CD11c, but they express the IL-3 receptor (CD123) [13]. These two DC subtypes also differ in their distribution throughout the body. MDCs are traveling from the bone marrow into the peripheral blood and/or out in peripheral tissues. The encounter of pathogens by tissue MDCs initiate their differentiation into mature DCs with the ability to migrate to lymphatic tissue and activate naïve T cells [11]. PDCs migrate from the bone marrow to the peripheral blood, but in contrast to MDCs, they relocate directly from the blood into secondary lymphoid tissue without encountering any antigen and PDC is the main producer of IFN-a in the body upon activation [13,14].

Several types of solid and blood cancers, such as pancreatic, breast, prostate, hepatocellular, lung, leukemia and squamous cell head and neck carcinomas, are accompanied by impaired function and reduced numbers of DCs [15-20]. This imbalance in the circulating DC pool is not just exclusively a finding in cancer, but is also observed in patients with chronic infections, such as HIV-1, hepatitis B, and hepatitis C, atopic dermatitis, and in autoimmune diseases, such as psoriasis arthritis, and rheumatoid arthritis [12,21-23].

The connection between these medical conditions is some degree of chronic inflammation, caused either by the tumor mass, infectious agents, or by autoreactive immune cells. The immune system serves to counteract the attack; which for a short period of time has beneficial consequences and under normal circumstances promotes the healing. However, it can be harmful when an inflammation becomes chronic and cause tumor escape from the immune surveillance [24,25], for instance as a result of dysfunctional immune cells.

In the present study, we investigated how the PDAC affect the MDCs and PDCs existing in peripheral blood. In addition, we wanted to study whether these populations of DCs return to normal after the tumor resection, which should be expected if the tumor was the only cause of the inflammation. We found that the PDAC, and other cancers located in the pancreas, such as biliary duct adenocarcinoma (BDAC), ampullary carcinoma (AC), and endocrine carcinoma (EC), all exerted systemic effects on the MDCs and PDCs, resulting in both reduced numbers and enhanced apoptosis. Incidentally, chronic inflammation of the pancreas, i.e. chronic pancreatitis, had the same effect on the DCs as the different tumors implicating chronic inflammation as a factor involved in this impairment. This could indicate that inflammation does not only directly support the development of the tumor, for instance by releasing growth stimulatory factors, but also indirectly by impairing the ability of DCs to activate immune response directed against the tumor. Of, note a preservation of the blood DCs compartment in PDAC patients seems to benefit the patients’ ability to manage the disease as PDAC patients with a survival over 2 years had significant higher levels of blood DCs compared to patients with less than one year survival.

## Methods

### Patients and controls involved in the study

Twenty ml heparinized peripheral whole blood samples were obtained from controls, at one occasion, and from patients at two time points, one week prior surgical removal of the tumor (Whipple resection) and 8-12 weeks after the surgery. The age matched controls were recruited randomly from department of Transfusion Medicine at Linköping University Hospital (Linköping, Sweden) and from the senior division of Linköping orienteering club. Subjects were consecutively recruited from the list of patients planned for pancreatic resection after preoperative radiological evaluation at Linköping University Hospital. The final diagnosis was histologically confirmed by two pathologists, independently investigating the samples. The patient group in this study referred to as billary duct adenocarcinoma (BDAC), are tumors histologically confirmed arising from the distal part of the billary duct located inside the pancreas. The patients with pancreatic disease did not receive chemo/radiotherapy during the time period of the pre or post blood sample collection and had no long term treatment with cortisone or NSAID. All samples were coded to protect the identities of the subjects participating in this study. The study protocol and patient consent documents were approved by the Regional Ethics committee in Linköping, Sweden (Dnr. M38-06). The PDACs were staged according to the 1997 International Union against Cancer classification (TNM = Tumor, Node, Metastasis).

### Separation of peripheral blood mononuclear cells

Peripheral blood mononuclear cells (PBMCs) were isolated from heparin treated whole blood by Ficoll-Paque PLUS (GE Healthcare, Uppsala, Sweden) density gradient centrifugation. The plasma layer was collected after the density centrifugation, aliquoted in cryogenic-tubes and stored at -70°C until analysis. The cellular interface containing the PBMCs was harvested and washed two times in Dulbecco's PBS without Ca^2+ ^and Mg^2+ ^(PAA Laboratories GmbH, Germany). The PBMCs were resuspended in PBS supplemented with 0.2% bovine serum albumin (PAA Laboratories GmbH, Germany) and the cell quantity and viability measured by staining with Trypan blue (Fisher Scientific, Västra Frölunda, Sweden). The PBMCs were diluted to 5 × 10^6 ^cells/ml and 5 × 10^5 ^cells were added to the wells of a 96-wells U-bottom plate for examining the DC frequency and phenotype (see below). The remaining cells were spun down and re-suspended in freezing media (fetal bovine serum containing 8% DMSO: (Sigma-Aldrich, Schnelldorf, Germany) and cryogenic preserved in a liquid nitrogen freezer.

### Flow cytometry monoclonal antibodies

Peripheral blood DC subsets were identified using FITC conjugated lineage (Lin) cocktail (CD3, CD14, CD16, CD19, CD20 and CD56), HLA-DR (PerCP), CD11c (APC), and CD123 (PE) monoclonal antibodies (mab) (Becton Dickinson, Stockholm, Sweden). Detection of apoptotic cells in peripheral blood was done by staining with Annexin V (APC) protein (Becton Dickinson, Stockholm, Sweden) in combination with FITC conjugated Lin cocktail, HLA DR PerCP and PE CD123 for PDCs or PE CD11c for MDCs.

### Flow cytometry acquisition and analysis

PBMCs (5 × 10^5^) were suspended in PBS supplemented with 0.2% BSA (FACS wash) and labeled with lineage cocktail, HLA-DR, CD11c, and CD123 mabs to detect MDCs and PDCs. The antibody straining was carried out at 4°C for 40 min. After the incubation unbound antibody was removed by spinning down the samples and replacing the supernatant with new FACS wash. This procedure was repeated 3 times. Detection of apoptotic DCs in the PBMCs was done by staining with Lin cocktail, HLA-DR, CD11c (for MDC) and Lin cocktail, HLA-DR, CD123 (for PDC) followed by incubation both sets with Annexin V protein for 15 min at 4°C. Four color flow cytometry was performed using a FACS Calibur flow cytometer (Becton Dickinson, San Jose, CA), analyzing 5 × 10^5 ^PBMCs for detection of apoptotic MDCs and PDCs and 2 × 10^5 ^PBMCs for determining the quantity of MDCs and PDCs. The acquired data were analyzed using the FLOW-JO software, v7.0 (Tree Star Inc, Ashland, OR).

### Cytokine array and ELISA

Plasma cytokine profiles were analyzed by Bio-Plex™ Human cytokine 27-plex panel (Biorad, Laboratories, Inc.). The plasma was thawed, processed, and analyzed as recommended by the manufacturer. The cytokine panel was analyzed using Luminex 100™ (Luminex, Inc) plate reader and data processed using the corresponding program. Concentrations of plasma PGE_2 _metabolites (Cayman Chemicals Company, Ann Arbor, USA) and TGF-β (EBioscience, Inc. San Diego, USA) were measured by EIA and ELISA, respectively, according to the manufacture protocols.

### Statistics

All groups were tested using Kruskal-Wallis one-way analysis of variance by ranks and when they were found significant followed by Mann-Whitney U test. P values < 0.05 were considered to be statistically significant. Correlation analysis of the data was performed using the Spearman rank correlation of nonparametric data.

## Results

### Characteristics of patients and controls

52 patients and 20 age matched controls were recruited to participate in this study. The cancer patients were divided according to cancer type, such as pancreatic duct adenocarcinoma (PDAC) (N = 25), ampullary carcinoma (AC) (N = 6), billary duct adenocarcinoma (BDAC) (N = 4) and endocrine carcinoma (EC) (N = 5). Furthermore, seven patients that underwent surgery for suspected tumor in the pancreas and turned out to have chronic pancreatitis (CP) (N = 7) were also included in this study. In six individuals the pancreatic tumor was deemed none resectable at laparotomy however they were included in the pre surgery group and termed none resectable pancreatic tumor (NRPT) (N = 6). Detailed characteristics of the different patient groups and controls are summarized in Table 1. The patients that fulfilled the criteria for surgical resection of the tumor mass donated peripheral blood around one week before (pre) resection and 8-12 weeks after (post) resection. The results obtained from individuals with PDAC and other pancreatic cancers and chronic pancreatitis were compared with twenty randomly selected age matched controls.

**Table 1:** Patient characteristics and tumor identification and staging

	Control	PDAC	NRPT	BDAC	AC	EC	CP
N Patients Pre surg.	20	25	6	4	6	5	7
N Patients Post surg.	N/A	16			6	4	6
Males	12	15	5	3	6	2	3
Females	8	10	1	1	0	3	4
Median age (range)	66 (49-86)	68 (47-78)	66 (51-74)	75 (65-78)	67 (53-75)	58 (46-67)	67 (59-72)
**TNM**							
1A					1	3	
1B		4		1	3	1	
2A		1				1	
2B		19		3	1		
3					1		
4		1					
**Differentiation**							
G1		5			2	2	
G2		13		1	3		
G3		7		3			
Not evaluated					1	3	

Pancreatic ductal adenocarcinoma (PDAC), None resectable pancreatic tumor (NRPT), billary duct adenocarcinoma (BDCA), ampullary carcinoma (AC), endocrine carcinoma (EC), chronic pancreatitis (CP), not applicable (N/A), Tumour, Node, Metastasis (TNM), number (N)

### Peripheral blood MDCs and PDCs are diminished in patients with pancreatic tumors, including PDAC, and chronic pancreatitis

**Figure 1:**
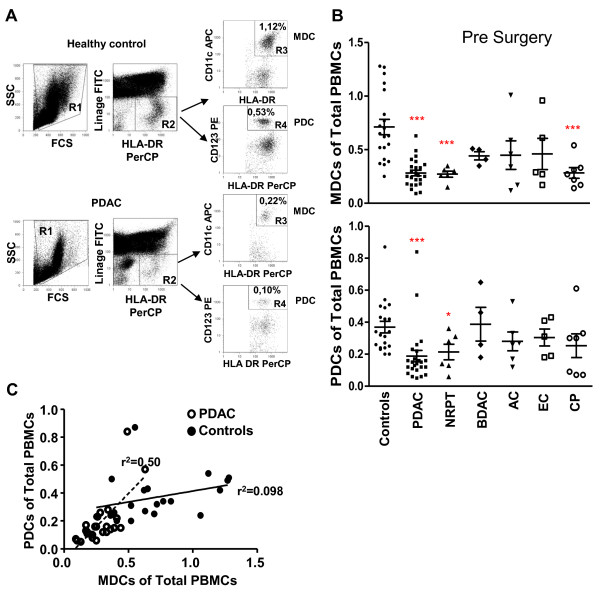
**Characterization and quantification of MDCs and PDCs**. **(A) **PBMCs isolated from individuals with pancreatic duct adenocarcinoma (PDAC) (1 week pre surgery) and healthy age matched volunteers were analyzed for MDCs and PDCs levels by flow cytometry. The PBMCs stained with different direct conjugated mabs to distinguish the MDC and PDC from the rest of the cells. The gating was set to exclude debris (R1) and second gate (R2) was set on Lineage (FITC) negative and HLA DR (PerCP) positive cells. The MDCs were identified as Lin-HLA DR+ CD11c+ cells (R3) and PDCs as Lin-HLA DR+ CD123+ cells (R4). Top panel shows the dot plots from a healthy volunteer and the bottom panel shows the dot plots from an individual with PDAC. **(B) **Percentage of MDCs (top panel) and PDCs (lower panel) prior tumor resection in healthy controls, PDAC, none resectable pancreatic tumor (NRPT), billary duct adenocarcinoma (BDAC), ampullary carcinoma (AC), endocrine carcinoma (EC), and chronic pancreatitis (CP). **(C) **Correlation of MDC and PDCs numbers (% out of PBMCs) between controls and PDAC. Control: Solid line and black circles r^2 ^= 0.50. PDAC: striped line and white circles r^2 ^= 0.098. Statistically significant differences between individuals with pancreatic disease and healthy controls are indicated as; * = p < 0.05, ** = p < 0.005, *** = p < 0.001

Several solid cancers display impaired function and numbers of blood DCs [15-20,26,27]. In the case of pancreatic cancer Yanagimoto et al found that both the circulating MDC numbers and their function were impaired [15,26]. We examined both MDCs and PDCs in peripheral blood from patients diagnosed with PDAC and compared this to levels found in other tumors located in the pancreas and age matched controls. The frequency of DCs was distinguished by flow cytometry by gating on HLA DR positive (gate R2) and lineage negative cells (i.e. to exclude other cell types). This population contains two DC subtypes, which can be distinguished from each other by gating on cells positive for CD11c which correlate to MDCs (gate R3) or for CD123 which correlate to PDCs (gate R4) (Figure 1A: representative data from one healthy control and one individual with PDAC). The frequency of MDCs and PDCs in blood was measured as the percentage of total PBMCs. This may not give the exact same levels as if analyzed as total numbers of PBMCs per ml blood but gives accurate values of the decrease in DCs occurring in individuals with pancreatic cancer compared to age matched healthy controls. Individuals with PDAC, NRPT, and CP all had significant decreased levels of MDCs compared to controls (Figure 1B), whereas the levels of PDCs were significantly reduced in PDAC, and NRPT as compared to controls (Figure 1B). Our age matched controls had equivalent levels of MDCs and PDCs as documented previously for this age group [28,29]. The MDCs constitute a larger population than the PDCs in a healthy individual. This relationship was altered in PDAC with a greater loss among MDCs than the PDCs, which brought about equal frequencies of these cells within the PBMCs (Figure 1C). In a few patients with PDAC the blood MDCs and PDCs were almost gone (Figure 1A; lower panel) indicating that this disease can exert systemic impairing effects on immune cells important for maintaining a functional immunity. Of note, we did not see any correlation between tumor differentiation grade or stage and the levels of blood DCs (data not shown). Our findings confirm the decreased frequencies measured in subjects with pancreatic cancers and other types of cancers [16-20]. The reasons why the MDCs and PDCs are more afflicted in PDAC patients than BDAC, AC, and EC are unclear, but could be due to the level of inflammation caused by the different tumors or to behavioral differences of the tumors.

### The blood MDCs and PDCs impairment persist in the majority of patients with PDAC and other cancers in the pancreas 12 weeks after tumor removal

**Figure 2:**
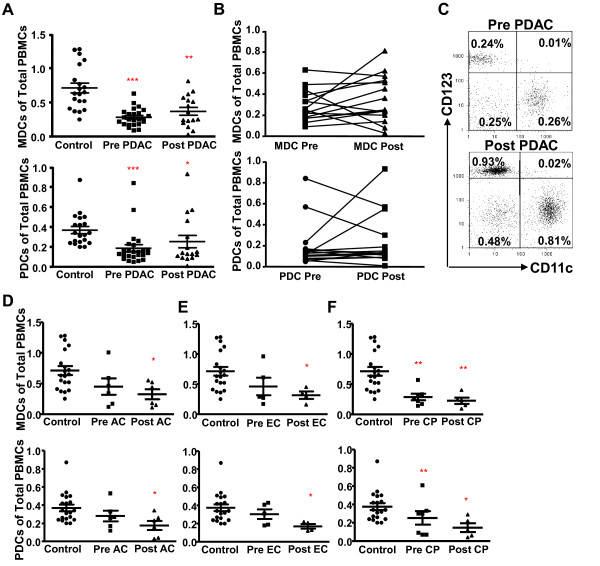
**Resection of pancreatic tumor mass restored the MDCs and PDCs in some subjects**. **(A) **Percentage of MDCs (top) and PDCs (bottom) of total PBMCs in healthy age match controls and pre and post tumor resection in individuals with pancreatic duct adenocarcinoma (PDAC) **(B) **Pre and post tumor resection levels of MDCs (top) and PDCs (bottom) in individuals with PDAC. **(C) **Dot plots showing the percentage of MDCs (CD11c+) and PDCs (CD123+) pre and post tumor resection in a subject with PDAC with fully recovered DC populations 12 weeks post surgery. Percent of MDCs (top) and PDCs (bottom) of total PBMCs in healthy age match controls and pre and post resection in individuals with **(D) **ampullary carcinoma (AC), **(E) **endocrine carcinoma (EC) and **(F) **chronic pancreatitis (CP). Statistically significant differences between individuals with pancreatic disease and healthy controls are indicated as; * = p < 0.05, ** = p < 0.005, *** = p < 0.001

Surgical removal of primary tumors can reverse tumor induced immunosuppression [30]. The recovery seems to take time as normalized levels of MDCs were only found in PDAC subjects that had been disease free 12 months after the tumor removal, whereas patients with recurrent disease or metastasis had no significant increase in these cells at this time point [26]. Of note, a significant decrease in blood DCs was seen initially six weeks post the breast cancer surgery [31]. To evaluate if the resection of the tumor in patients with PDAC or other cancers in the pancreas restored or lowered the blood DC levels, the levels of MDCs and PDCs were assessed 8 to 12 weeks after resection. The percentage of MDCs and PDCs were significantly increased in some of the subjects after tumor resection, whereas others surprisingly had even lower levels than before the tumor resection (Figure 2A, B, C, D, E, and 2F). The amounts of MDCs increased more than 30% in 8 of the individuals with PDAC (Figure 2B) whereas only 3 individuals had a more than 30% increase in PDCs (Figure 2B). Only **3 **patients with PDAC, among all patients and type of cancer examined, had total recovery of MDCs and PDCs post surgery (Figure 2C; example of patient with full recovery) and this correlated with decreased levels of inflammatory factors seen post surgery. Surprisingly, the tumor removal even provoked a further decrease in MDCs (n = 3) and PDCs (n = 5) in some of the subjects examined in this study. Moreover, this decrease post surgery was also seen for patient with AC, EC, and CP (Figure 2D, E, and 2F). Explanations for why the DC subsets did not return to normal or even decreased post surgery could be due to, the high recurrence rate seen among surgical treated PDAC patients, that the surgically procedure itself induced a setting with more inflammation or that the initial inflammation had not been cleared. The immune system by it self should not require longer time to recover as the levels and functions of DCs in most of HIV-1 infected individuals starts to recover soon after the viral load is abolish [21]. In conclusion, our results point to an induction of a systemic impairment of the immune system and its cells, i.e. blood MDCs and PDCs, by the tumor mass and/or fibrotic mass. This impairment was reversed in some individuals when the primary tumor was removed and the inflammation resolved.

### Increased numbers of circulating Lin-HLA DR+ CD123- CD11c- cells in peripheral blood from individuals with ductal pancreatic adenocarcinoma

**Figure 3:**
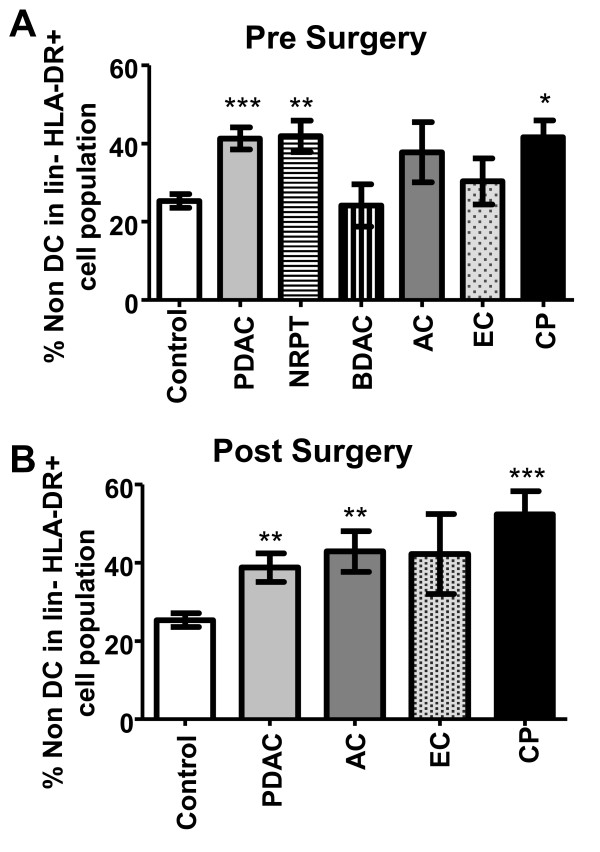
**The non DC in lin-HLA DR+ cell population is increased in individuals with different types of pancreatic cancers and chronic pancreatitis**. Percentage of non DC (lin-HLA DR+ CD11c-CD123-) within the lin-HLA DR+ cell population in healthy age match controls and pancreatic duct adenocarcinoma (PDAC), none resectable pancreatic tumor (NRPT), billary duct adenocarcinoma (BDAC), ampullary carcinoma (AC), endocrine carcinoma (EC), and chronic pancreatitis (CP) prior surgery **(A) **and after surgery **(B)**. The proportion of non DC was estimated as the mean percentage of the total amount of lin-HLA-DR+ cells. Statistically significant differences between individuals with pancreatic disease and healthy controls are indicated as; * = p < 0.05, ** = p < 0.005, *** = p < 0.001.

Accumulation of lineage- (lin-) HLA DR+CD11c-CD123- blood cells (non DC) coincides with a reduction in the CD11c+ DC and/or CD123+ DCs in subjects with breast cancer, prostate cancer, and malignant glioma [32]. We noticed that absolute numbers of the non DCs were comparable between controls and the different pancreatic cancer subjects with the exception for BDAC, which had a decrease in the total numbers of these cells (data not shown). Of note, even if the absolute numbers of these cells remained the same did the composition of the lin-HLA DR+ population change with significantly increased frequency of non DCs, and decreased PDCs and MDCs for PDAC, NRPT, AC, EC, and CP (Figure 3A). The resection of the tumor mass did not diminish the elevated levels of non DCs in the lin- HLA DR+ cell population (Figure 3B). The type of cell or cell progenitor that the non DC population corresponds to needs further evaluation, however we can exclude leukocytes such as normal monocytes, macrophages, B cells, T cells, NK cells, neutrophils, eosinophils, basophils, MDCs, and PDCs. Interestingly, Pinzon-Charry et al showed that this non DC population increases with metastatic disease compare to local disease and controls suggesting that there is an association with disease augmentation [32]. The reasons for why this cell population did not decline to a higher extent in our cohort could be due to their disease status or too short time frame from the surgery to have restored the blood composition.

### Elevated levels of apoptotic blood MDCs and PDCs in PDAC and chronic pancreatitis

**Figure 4:**
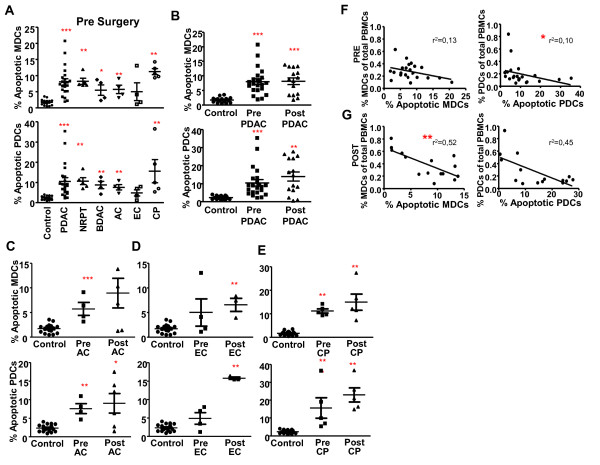
**Increased spontaneous apoptosis among the MDCs and PDCs in individuals with PDAC and other types pancreatic cancers**. **(A) **Percentage apoptotic MDCs (top panel) and PDCs (lower panel) of total MDCs and PDCs prior tumor resection in controls, pancreatic duct adenocarcinoma (PDAC), none resectable pancreatic tumor (NRPT), billary duct adenocarcinoma (BDAC), ampullary carcinoma (AC), endocrine carcinoma (EC), and chronic pancreatitis (CP). Percent apoptotic MDCs (top) and PDCs (bottom) of total MDCs and PDCs in healthy age match controls and pre and post tumor resection in individuals with PDAC **(B)**, AC **(C)**, EC **(D) **and CP **(E)**. Correlation between percent apoptotic MDCs and level of MDCs (left) and correlation between percent apoptotic PDCs and level of PDCs (right) in individuals with PDAC pre tumor resection **(F) **and post tumor resection **(G)**. The numbers of Annexin V positive MDCs and PDCs were measured as the percentage of Annexin V+ cells out of the total amount of MDCs or PDCs. Statistically significant differences between individuals with pancreatic disease and healthy controls are indicated as; * = p < 0.05, ** = p < 0.005, *** = p < 0.001.

Immune cells circulating in peripheral blood such as MDCs, PDCs, and T cells in individuals with breast cancer, melanoma and head and neck cancer are affected by the solid tumors as they display an increased spontaneous programmed cell death, i.e. apoptosis [33-35]. We used the Annexin V protein to detect apoptotic cells in the PBMCs from healthy controls or individuals with PDAC, other cancers in the pancreas and CP (Figure 4A). The amounts of apoptotic MDCs and PDCs were significantly higher pre surgery in all pancreatic cancers besides for EC (Figure 4A, B, C, D, and 4E). The levels of apoptosis in MDCs and PDCs from PDAC patients post surgery were similar to the levels seen pre surgery. In contrast, apoptosis of PDCs and MDCs increased after surgery in EC (Figure 4D) and AC showed the same tendency (Figure 4C). Notably, the individuals with CP had the highest level of MDCs and PDCs apoptosis both pre and post surgery (Figure 4E). The reasons behind the increase in apoptotic cells post surgery could be enhanced and/or changed composition in inflammatory factors during the healing process. We found a significant negative correlation of the levels of PDCs found in peripheral blood prior and after surgery with the levels of apoptosis among these cells from individuals with PDAC (Figure 4F, and 4G), whereas this correlation for MDCs only was significant for the post surgery samples (Figure 4F, and 4G). This finding may indicate that the decrease in DC in individuals with PDAC and other types of diseases in the pancreas could be due to increased apoptosis.

### Elevated PGE2 and CXCL8 in plasma from individuals with PDAC

**Figure 5:**
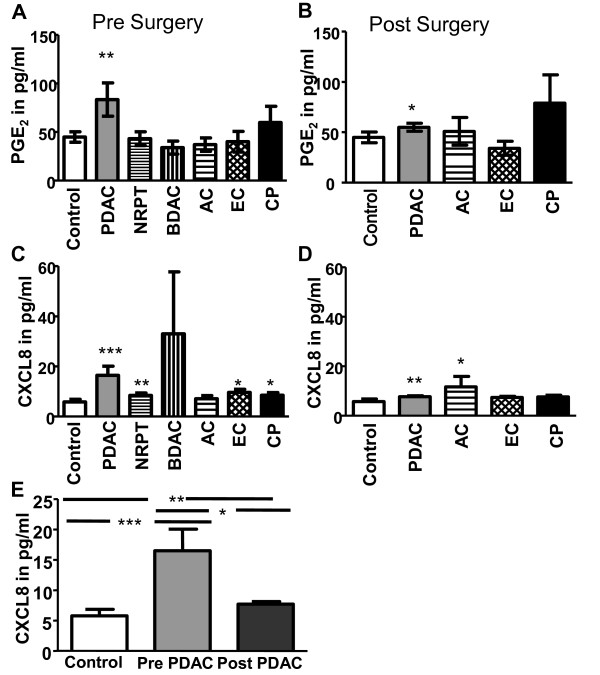
**Elevated levels of inflammatory cytokines in individuals with pancreatic duct adenocarcinoma**. PGE_2 _levels in plasma from pancreatic duct adenocarcinoma (PDAC), none resectable pancreatic tumor (NRPT), billary duct adenocarcinoma (BDAC), ampullary carcinoma (AC), endocrine carcinoma (EC), and chronic pancreatitis (CP) pre **(A) **and post **(B) **tumor resection compared to levels in healthy age matched controls. CXCL8 levels in plasma from PDAC, NRPT, BDAC, AC, EC, and CP pre **(C) **and post **(D) **tumor resection compared to levels in healthy age matched controls. CXCL8 levels in plasma from PDAC pre and post **(E) **tumor resection compared to levels in healthy age matched controls. Statistically significant differences between individuals with pancreatic disease and healthy controls are indicated as; * = p < 0.05, ** = p < 0.005, *** = p < 0.001.

Results points to that the COX-2 product PGE_2 _mediated PDAC cellular invasiveness through an ERK/Ets-1-dependent induction of MMP-2 expression and activity [36]. We found here significantly elevated PGE_2 _levels in individuals with PDAC both pre- and post surgery even if the levels decreased after surgery (Figure 5A, B, C, and 5D). The PGE_2 _levels were not elevated pre or post surgery in the other pancreatic tumors, i.e. BDAC, AC, and EC (Figure 5A, B, C, and 5D). Incidentally, CP had also elevated PGE_2 _levels but was not found statistically significant (Figure 5A). Other immune regulatory factors have been found elevated, i.e. IL-10, which have been described as negative indicator for survival of patients with different types of cancers. IL-10 in serum from patients with hepatocellular carcinoma correlates with decreased number of DCs and immature DCs subsets [16]. However, we could not observe any significant increase in IL-10 levels in our samples compared to controls (data not shown). Similar inflammatory components and downstream effectors have been found to be elevated in CP and PDAC, such as CXCL8, an activator of inflammatory NF-kB cascade and associated with tumorigenesis by promoting angiogenesis and metastasis [37]. In addition, mRNA CXCL8 are over expressed in ~80% of PDAC tissue samples compared to normal surrounding tissue [38]. The individuals with PDAC, NRPT, EC, and CP had significantly elevated levels of serum CXCL8 pre surgery (Figure 5C). Post surgery, PDAC and AC were the only diseases that had elevated levels of CXCL8 (Figure 5D). Interestingly, the removal of the tumor mass or suspected to be tumor mass (massive fibrosis) lowered the CXCL8 levels for PDAC, EC, and CP but only significantly for PDAC (P = 0.008) (Figure 5E). We investigated the levels of TGF-β and several other inflammatory factors in our samples and could not see any significant increase or decrease of these factors in any of the individuals with different tumors in the pancreas (data not shown). In summary, our findings point to systemic effects exerted by the presence of solid tumor and chronic inflammation in the pancreas that create elevated levels of immunoregulatory soluble factors such as CXCL8 in peripheral blood.

### Long time PDAC survivors are presented with more circulating DCs than short time survivors

**Figure 6:**
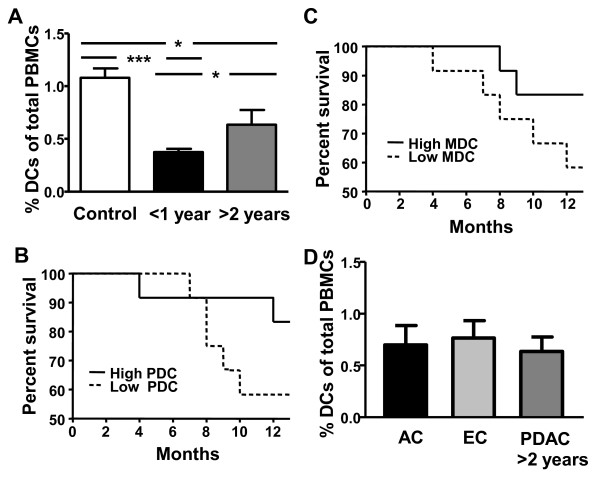
**Long-time survivors have more DCs compared to short-time survivors**. **(A) **Percent DCs of total PBMCs in healthy age match controls and pre tumor resection in PDAC patients surviving less than one year (short-time survivor) and more than two years survival (long-time survivor). **(B and C) **Comparison of the one year survival between the PDAC patient group with the lowest (N = 12) and highest (N = 12) levels of MDCs (≤0.25% and >0.25%) and PDCs (≤0.14% and >0.14%), respectively. **(D) **Percent DCs of total PBMCs in PDAC patients with more than two years survival (long-time survivor) and in patients with ampullary carcinoma (AC) and endocrine carcinoma (EC) patients. Statistically significant differences between individuals with pancreatic disease and healthy controls are indicated as; * =p < 0.05, *** = p < 0.001.

The total amount of blood DCs pre surgery were compared between patients with PDAC surviving less than one year (n = 7) (short time survivors) and patients surviving more than two years (n = 6) (long time survivors) after surgery. The rest of our patient cohort had less time than 2 years since their surgery so they did not fit these categories for long and short time survivors. Data from our patient cohort showed a significant difference in the levels of blood DCs between the group with short (0.38%) and long time (0.64%) survival (Figure 6A). Of note, both groups had decreased amount of total blood DCs compared to healthy controls (1.08%) (Figure 6A). To investigate the importance of the amount of circulating MDCs and PDCs for patient survival, the PDAC patients were divided into two groups; one group with the 12 lowest and one group with the 12 highest levels of blood MDC ((≤0.25% and >0.25% MDCs) and PDC (≤0.14% and >0.14% PDCs)). The low MDC and PDC groups had a similar one year survival rate of 58%, compared to 83% for the high MDC and PDC groups (Figure 6B, and 6C).

Some of the other tumors found in pancreas, i.e. ampullary and endocrine carcinoma have higher 5 years survival rates (60%) [39,40] and both carcinoma groups were found with similar DC levels pre surgery (0.73% and 0.76%, respectively) as the patients with PDAC with long time survival (0.64%) (Figure 6D). Hitherto, we have not found any correlations between the patients' survival times and the level of inflammatory factors CXCL8 and PGE2 in their peripheral blood. Our data show the connection of longer survival time and level of DCs in the blood compartment in patients with PDAC and this need to be addressed in a bigger cohort of patients and as far as we know has this not been shown previously and need further elucidation.

## Discussion

DCs have been recognized as the main initiators of the adaptive immune response and play the pivotal role of tumor surveillance in healthy individuals. The number of peripheral blood DCs appears to be decreased in several types of cancer, including pancreatic cancer [15-20,31,32]. An opposing finding has been shown for levels of DC in individuals with melanoma, which had increased levels of both MDCs and PDCs with the highest in stage I disease but even stage IV had elevated levels [41]. Findings from several types of solid cancers imply that anti-tumor immunity may be related to the number and/or functions of DCs [15-17,19,20,26]. Our results indicate that there is a significant decrease in the amount of MDCs and PDCs in peripheral blood from patients with different types of pancreatic cancers, including PDAC, but also for chronic inflammation in pancreas, i.e. chronic pancreatitis. A decrease in the MDC and an increase in the PDC subsets have previously been described for PDAC [15]. The latter observation are contradicting the results we have obtained in the present study for PDAC and other pancreatic cancer as well as in previous findings for different adenocarcinoma [17,20,31]. These differences are probably due to the definition of PDCs as CD11c-/lin-/HLA DR+ without using any specific marker for PDCs, which will include a non PDC population besides the PDCs [15].

The peripheral Lin- HLA DR+ cell population contains MDCs, PDCs, and cells that are lin^-^HLA-DR^+^CD123^-^CD11c^- ^(non DC). Our findings show a significantly increased frequency of non DCs among the Lin- HLA DR+ population in patients with PDAC. This corroborates a previous study by Pinzon-Charry et al displaying an increase in this population in breast cancer, prostate cancer, and malignant glioma [32]. These lin- HLA-DR+CD11c-CD123- cells might be a specific pre DC population but they have less efficient antigen presenting function and generated an inadequate immune response compared to MDCs and PDCs [32]. Whether these cells are MDCs and PDCs, that have impaired phenotype and functions due to the systemic effect by the disease, or if they comprise a separate cell population of different origin or a progenitor for one of the blood lymphocyte will need further elucidation. The increased proportion of this non-DC population among HLA DR+ cells appears when the MDC and PDC numbers decreases. Conversely, the diminished levels of DCs could be due to the enhanced apoptosis seen in both DC subsets in individuals with PDAC and other types of cancers in the pancreas. There was a negative correlation between levels of PDC and levels of apoptotic cells, which supports the involvement of program cell death as one component in the decrease. Of note, the frequency of apoptotic DCs remained the same or increased after the surgical removal of tumors (PDAC, EC, and AC) or inflamed tissue (CP) and the reason for the increased apoptosis could be enhanced inflammation secondary to the surgical procedure or the healing process. Increased apoptosis in the blood DCs have previously not been described for different types of pancreatic cancers or chronic pancreatitis, whereas MDCs, PDCs, and T cells in individuals with breast cancer, melanoma, and head and neck cancer have been shown to have increased levels of apoptosis [33-35]. Taken together, these data prove that the solid tumor exert systemic modulatory effects on the immune system.

Dysfunctional DCs exist in many cancers including PDAC, where impairment of T cell stimulatory function among the MDCs has been detected [15]. Another example is breast cancer, which besides decreased T cell stimulatory ability, exhibits decreased IL-12 and increased IL-10 production [17]. It's likely that individuals with PDAC, and other pancreatic cancers, with decreased amounts of MDCs and PDCs and increased level of the non DCs provide a setting were the DCs in blood and tissue are inadequate to initiate a sufficient immune response against the tumor. Surgical removal of primary tumors can reverse the tumor induced immunosuppression [30], which points to the tumor and surrounding stroma cells as general sources of inflammation inflicting impairment in the MDCs and PDCs. So, when resecting this inflammatory catalyst the immune system should get a second chance to reorganize and possibly kill remaining tumor cells. Unfortunately, this seems to have taken place only in a minority of the PDAC patients in a timely manner but on the other hand, these patients seem to have a complete recovery of the circulating DC populations. This failure to normalize MDC in subjects with PDAC was also seen in a recent study 2.5 to 13 weeks post surgery [26] and Pinzon-Charry et al even found a further decrease in blood DCs six weeks post breast cancer surgery [31].

Tumors have been referred to as wounds that never heal, due to their ability to create an inflammatory microenvironment [42]. Resection of the tumor should principally rescue the body from this chronic wound, but it seems likely that the healing process after surgery by itself contributes to the impairment of the circulating DCs as we found increased apoptosis in all pancreatic cancer groups and CP and even a further decrease in the frequency of DCs in many of subjects. Patients with early breast cancer disease showed minimally reduced DC levels at diagnosis but displayed a prolonged period (one year) of marked DC suppression after tumor resection [31]. For the majority of PDAC patients this time frame is too long since the disease gives them a shorter mean survival span than it takes for the DCs to recover.

Production of residual inflammatory and/or additional factors induced and produced during the healing process could be blamed for the sustained negative effect exerted on the DCs. Cyclin E1, epithelial growth factor (EGF) [37], IL-6, CXCL8, IL-10, and IL-1RA have all been shown to be elevated in individuals with pancreatic cancer [43,44] and high IL-6 or IL-10 levels correlated to poor survival [43]. Over expression of CXCL8 mRNA was found ~80% of PDAC tissues compared to corresponding normal surrounding tissue [38] and CXCL8 was also present in CP [37]. We found elevated levels of plasma CXCL8 in PDAC, EC, and CP pre surgery and that CXCL8 decreased after tumor resection. Of note, it has been shown that CXCL8 in PDAC is associated with tumor genesis by promoting angiogenesis and metastasis [38] but not with survival [43]. Furthermore, CXCL8 is a chemoattractant that will attract many different cell types expressing CXCR1 or CXCR2 including DCs [45] and the presence of CXCL8 in blood may affect their ability to exit the blood stream and affect their viability. Our findings do not show correlation between the increased plasma levels of CXCL8 and the amount of MDCs and PDCs, neither to the level of apoptosis or patient survival. The levels of CXCL8 decreased significantly in the blood from PDAC patients post surgery but this had no direct effects on the post surgery levels of MDCs or PDCs. Taken together, our findings indicate that CXCL8 is not directly involved in the depletion of blood DCs in PDAC or other pancreatic tumor patients but do not exclude that the recovery of DCs may take longer time than the normalization of inflammatory factors, such as CXCL8, in blood.

COX-2 enzyme expression is found in several cancers including PDAC and it's involved in cancer differentiation, apoptosis, metastasis, and angiogenesis [46-50]. We found the COX-2 metabolite PGE_2 _to be elevated significantly only in PDAC patients and tumor resection lowered the levels to almost normal. Increased levels of PGE_2 _in plasma seem to be a specific feature for PDAC and a possible marker for distinguish PDAC from other tumors in the pancreas, but further test must be performed. Of note, PGE_2 _is known to affect the DC function including, antigen presentation, maturation, and T cell activation [51] and this could be true for the MDCs and PDCs in PDAC. Furthermore, patients with chronic pancreatitis had also elevated levels but the PGE2 did not normalize after surgery. The increased plasma levels of PGE_2 _did not correlate to the amount of MDCs and PDCs, level of apoptosis or patient survival. However, the high expression of PGE_2 _and CXCL8 in PDAC patients could reflect the severity of this tumor compared to other pancreatic tumors [39,40].

Our findings show that patients surviving more than 2 years are represented with more circulating DCs than short time survivors. Some of the other tumors in the pancreas, i.e. ampullary and endocrine carcinoma have high 5 years survival rates (60%) [39,40] and both groups were found with similar DC levels presurgery (0.73% and 0.76%, respectively) as the patients with PDAC with long time survival (0.64%). Moreover, the one year survival was 83% in the group of PDAC patients with the highest MDC and PDC levels compared to only 58% in the group of PDAC patients with the lowest MDC and PDC levels. These findings indicate that the total levels of DCs pre surgery could predict patient survival. Moreover, our findings indicate that patients with un-affected blood DC subsets pre and post, or with normalized DC numbers post surgery seems to have a survival benefit compared to individuals with impaired numbers of DCs. The few patients fitting these characteristics had a survival over 30 months post surgery which is above the mean survival after pancreaticoduodenectomy for PDAC which is between 14 to15 months [52-54] indicating the importance of an intact blood DC compartment.

## Conclusions

Individuals with cancer or chronic inflammation in the pancreas have blood DCs characterized by both reduced numbers and enhanced apoptosis. The post-surgery follow up revealed a DC compartment still impaired in the vast majority of individuals examined, but a few individuals with cancer had normalized the circulating DC compartment. The preservation of the blood DCs compartment in PDAC patients seems to benefit their ability to control the disease and survival. If the inability to restore the DC compartment depends on irreversible effects exerted by the PDAC or a slow recovery of the immune system in this type of pancreatic cancer will need further evaluation seeing that our findings indicate that the levels of MDCs and PDCs correlate with survival.

## Competing interests

The authors declare that they have no competing interests.

## Authors' contributions

VT carried out experiments, analyzed data and helped writing this manuscript. PS, AS, KB, and DS contributed with ideas, crucial patients and/or analyzed data, and edited the paper. UF, SF and KM contributed with ideas and edited the paper. ML conceived, designed, and supervised the study and wrote the manuscript. All authors read and approved the final manuscript.

## Pre-publication history

The pre-publication history for this paper can be accessed here:

http://www.biomedcentral.com/1471-2407/10/87/prepub
